# O-GlcNAcylation of RPA2 at S4/S8 antagonizes phosphorylation and regulates checkpoint activation during replication stress

**DOI:** 10.1016/j.jbc.2024.107956

**Published:** 2024-11-02

**Authors:** Jianxin Zhao, Guangcan Shao, Xiaoxuan Lu, Zhuan Lv, Meng-Qiu Dong, Xiaoqian Liu, Jing Li

**Affiliations:** 1 Beijing Key Laboratory of DNA Damage Response and College of Life Sciences, Capital Normal University, Beijing, China; 2 National Institute of Biological Sciences, Beijing, China; 3 State Key Laboratory of Microbial Technology, Shandong University, Qingdao, China

**Keywords:** RPA2, O-GlcNAc, replication stress, checkpoint, phosphorylation

## Abstract

O-linked N-acetylglucosamine (O-GlcNAc) is the most abundant mono-saccharide modification occurring in the cytoplasm, nucleus, and mitochondria. The recent advent of mass spectrometry technology has enabled the identification of abundant O-GlcNAc transferase (OGT) substrates in diverse biological processes, such as cell cycle progression, replication, and DNA damage response. Herein we report the O-GlcNAcylation of Replication Protein A2 (RPA2), a component of the heterotrimeric RPA complex pivotal for DNA metabolism. We found that RPA2 interacts with OGT, and a topoisomerase II inhibitor, etoposide, diminishes the association. Using higher-energy collisional dissociation mass spectrometry, we mapped RPA2 O-GlcNAc sites to be Ser-4/Ser-8, which are well-known PIKK-dependent RPA2 phosphorylation sites involved in checkpoint activation upon replication stress. We further demonstrated that Ser-4/Ser-8 O-GlcNAcylation antagonizes phosphorylation and impairs downstream Chk1 activation. Moreover, RPA2 O-GlcNAcylation sustains H2AX phosphorylation upon etoposide treatment and promotes inappropriate cell cycle progression, indicative of checkpoint defects. Our work not only unveils a new OGT substrate, but also underscores the distinct roles of OGT in replication *versus* replication stress.

O-linked β-N-acetylglucosamine (O-GlcNAc) glycosylation occurs on Ser/Thr residues and is a reversible modification. It is catalyzed by the sole O-GlcNAc transferase (OGT), and removed by the sole eraser O-GlcNAcase (OGA) (1, 2). Currently, it is found to decorate more than 5000 proteins in the cytosol, nucleus, and mitochondria. O-GlcNAcylation frequently crosstalks with other post-translational modifications (PTMs), *e.g.*, phosphorylation (2), ubiquitination (3), Poly(ADP-ribosyl)ation (PARylation) (4) and regulates various biological processes, including transcription, translation, and immune response (1, 2).

Accumulating evidence suggests that O-GlcNAc plays a fundamental role in replication, mitosis, and DNA damage response (5, 6, 7, 8). For instance, OGT binds to and modifies the Minichromosome Maintenance (MCM) 2, 3, 6, and 7 subunits in the hexameric MCM helicase complex, increasing the stability of MCM–MCM ring interaction and enhancing their binding to chromatin (9). Additionally, histone H4S47 O-GlcNAcylation has been found to regulate replication origin activation (10). Furthermore, Topoisomerase IIα (TOP2A) is O-GlcNAcylated, which promotes malignant tumor progression in breast cancer and resistance to adriamycin by stimulating the binding of TOP2A to chromatin and its topoisomerase activity (11). Moreover, human flap endonuclease 1 (FEN1) is also O-GlcNAcylated. FEN1 O-GlcNAcylation at Ser352 not only disrupts the interaction of FEN1 with proliferating cell nuclear antigen at the replication foci, but also leads to altered cell cycle, defects in DNA replication, accumulation of DNA damage, and enhanced sensitivity to DNA damage agents (12). The Poly(ADP-ribose) glycohydrolase (PARG) is the eraser of protein PARylation and is found to be critical for replication (13). PARG is also O-GlcNAcylated, which elevates its binding to the chromatin (4). Because of the chromatin association of OGT, it is reasonable to speculate that there are many more OGT substrates involved in DNA replication.

O-GlcNAcylation also exerts its effect on DNA translesion synthesis (TLS) (5, 6), an error-prone pathway that replicates lesion-containing DNA. DNA polymerase η (Polη), a low-fidelity DNA polymerase, utilizes the damaged DNA as templates to restart DNA synthesis upon bulky DNA lesions. O-GlcNAcylation of Polη promotes its polyubiquitination at Lys462 and subsequent removal of Polη from replication forks, leading TLS to bypass cisplatin-induced lesions and causing increased cellular sensitivity to cisplatin (14). Upstream of Polη is the ubiquitin E3 ligase Rad18, which functions in both TLS and homologous recombination (HR). Rad18 is O-GlcNAcylated at Ser130/Ser164/Thr468, which is pivotal for its recruitment for DNA damage sites, essential for Polη focus formation and UV sensitivity (15).

The Replication Protein A (RPA) complex plays essential roles in DNA replication, and recombination, and also acts as a key sensor in the DNA damage response (16). It is a heterotrimeric complex composed of the RPA1 (RPA70), RPA2 (RPA32), and RPA3 (RPA14) subunits that bind to single-stranded DNA (ssDNA) with high affinity and protect it against breakage (17).

The RPA-ssDNA platform is subject to many PTMs. RPA2, in particular, is extensively phosphorylated during the cell cycle and in response to DNA damage. RPA2 has an intrinsically disordered N-terminus, an oligonucleotide/oligosaccharide-binding (OB) fold, and a flexible C-terminal winged helix (WH) domain. Upon DNA damage, ataxia-telangiectasia mutated- and Rad3-related (ATR) phosphorylates RPA2 at Ser33, and the phospho-deficient RPA2 mutant decreased DNA synthesis and increased accumulation of ssDNA after hydroxyurea treatment, suggesting that RPA2 phosphorylation at stalled forks alleviates replication stress (18). RPA2 Ser33 phosphorylation stimulates the subsequent phosphorylated by cyclin-dependent kinase (CDK) at Ser23/Ser29 (19, 20). Additionally, Ser23/Ser29 phosphorylation also plays a role in DNA damage, as the S23A/S29A mutant shows a decrease in the persistence and intensity of γH2AX foci after camptothecin or bleomycin treatment (21). These phosphorylation events facilitate DNA-dependent protein kinase catalytic subunit (DNA-PKcs)-dependent phosphorylation of RPA2 at Ser4/Ser8, which regulates replication fork restart and late origin firing in response to replication stress and suppresses replication stress-induced mitotic catastrophe (17, 18). The versatility and variability of RPA2 phosphorylation thus confers its functional diversity in multiple DNA metabolic pathways.

Herein, we show that RPA2 is O-GlcNAcylated. Using higher-energy collision dissociation (HCD) mass spectrometry (MS), we mapped two O-GlcNAc sites, Ser4 and Ser8. Mutagenesis studies demonstrate that the S4A/S8A mutations significantly downregulated O-GlcNAcylation levels. Using phospho-specific antibodies, we demonstrate that RPA2 O-GlcNAcylation antagonizes phosphorylation at S4/S8 and inhibits Checkpoint kinase 1 (Chk1) activation. Taken together, our work suggests that RPA2 O-GlcNAcylation antagonizes phosphorylation and regulates checkpoint activation during replication stress.

## Results

### RPA2 interacts with OGT

**Figure 1:**
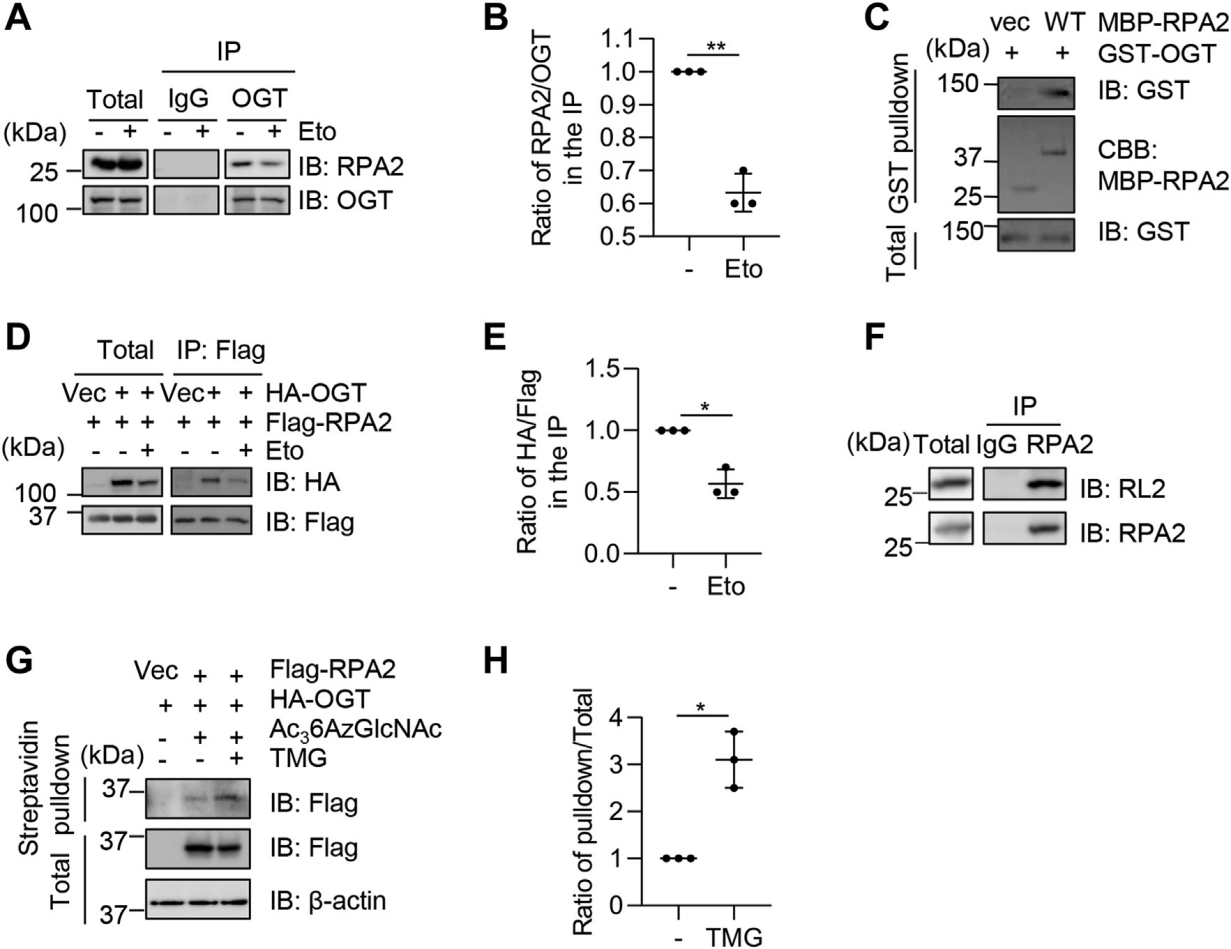
**RPA2 interacts with OGT.***A*, 293T cell lysates were immunoprecipitated with anti-OGT antibodies and immunoblotted with the indicated antibodies. *B*, quantitation of (*A*). *C*, recombinant MBP-RPA2 and GST-OGT proteins were incubated and subject to pulldown assays. *D*, 293T cells were transfected with Flag-RPA2 and HA-OGT. The cell lysates were subject to immunoprecipitation and immunoblotting with the antibodies indicated. *E*, quantitation of (*D*). *F*, cell lysates were immunoprecipitated with anti-RPA2 antibodies and immunoblotted with RL2 antibodies. *G*, cells were transfected with Flag-RPA2 and HA-OGT plasmids, then treated with 200 μM Ac_3_6AzGlcNAc and 5 μM Thiamet-G (TMG). *H*, quantitation of (*G*). Quantitation in *B*, *E*, *H* was done with a Student’s *t* test. ∗ indicates *p* < 0.05; ∗∗ indicates *p* < 0.01.

As O-GlcNAcylation has been shown to participate in DNA damage response (6), and RPA2 plays an important role in DDR (16), we wondered whether OGT could O-GlcNAcylate RPA2. To this end, we first examined the potential biochemical interaction between OGT and RPA2. Cell extracts were immunoprecipitated (IPed) with anti-OGT antibodies and the immunoprecipitates were immunoblotted (IBed) with anti-RPA2. RPA2 was found to co-IP with OGT (Fig. 1*A*). Interestingly, we discovered that the interaction between OGT and RPA2 was reduced after etoposide (Eto) treatment (Fig. 1, *A* and *B*). Then we purified MBP-RPA2 and GST-OGT and found MBP-RPA2 could pull down GST-OGT (Fig. 1*C*). To investigate whether RPA2 interacts with OGT exogenously, we co-transfected 293T cells with HA-OGT and Flag-RPA2 plasmids and performed co-immunoprecipitation (coIP) assays with the anti-Flag antibody (Fig. 1*D*). RPA2 was also found to co-IP with OGT and Eto diminished the interaction between OGT and RPA2 (Fig. 1, *D* and *E*).

We then investigated whether RPA2 is O-GlcNAcylated. Cell extracts were immunoprecipitated with anti-RPA2 antibodies and the immunoprecipitates were immunoblotted with anti-RL2 antibodies. The immunoprecipitated RPA2 showed an apparent O-GlcNAc band (Fig. 1*F*). Click chemistry was also utilized to examine RPA2 O-GlcNAcylation (Fig. 1, *G* and *H*). We observed that Thiamet-G (TMG) treatment increased the O-GlcNAcylation levels of RPA2 (Fig. 1, *G* and *H*). These results suggest that RPA2 interacts with OGT and is O-GlcNAcylated.

### RPA2 is O-GlcNAcylated at Ser-4/Ser-8

**Figure 2:**
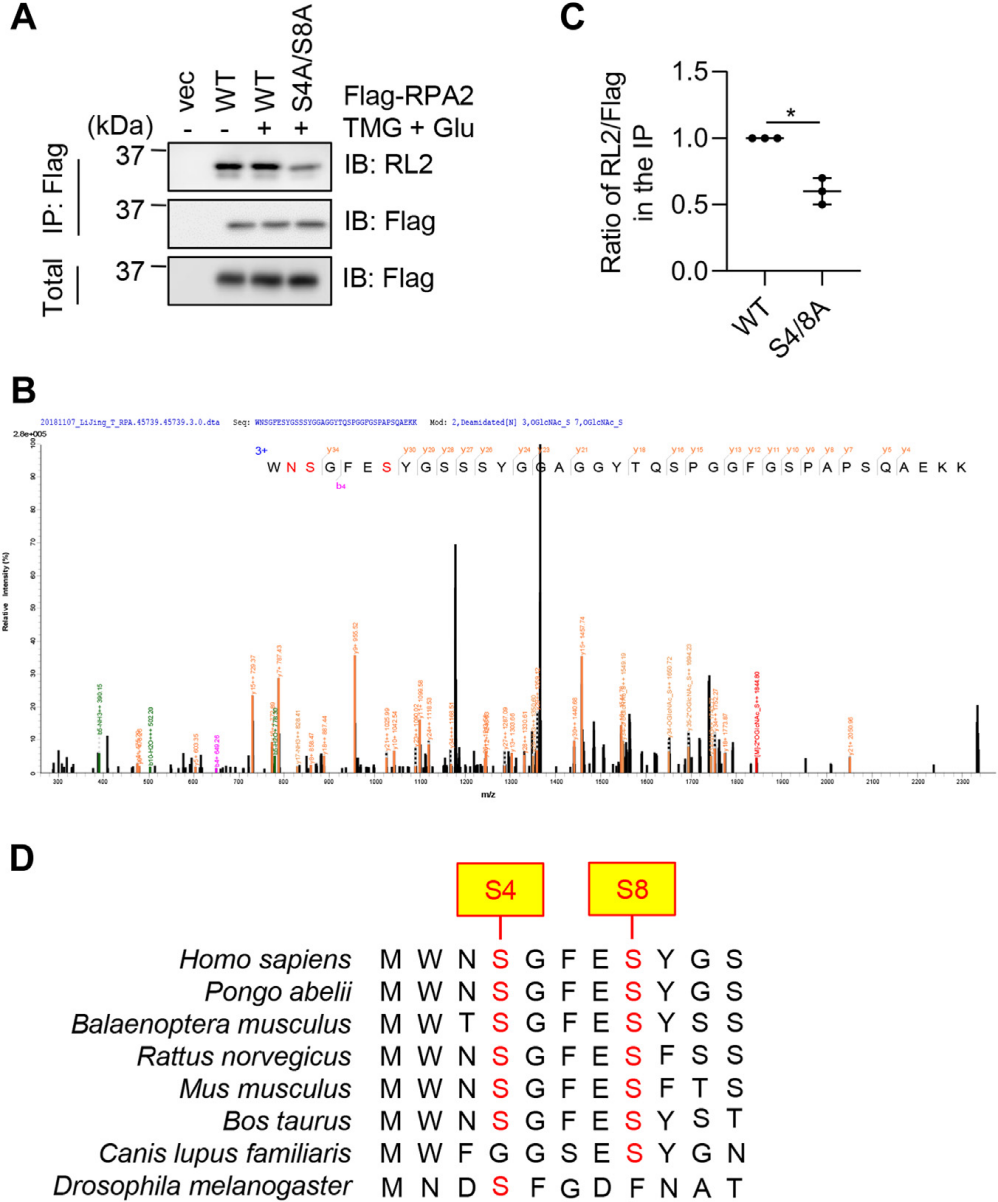
**RPA2 is O-GlcNAcylated at S4/S8.***A*, cells were transfected with vector, Flag-RPA2, S4/S8A plasmids, then treated with the OGA inhibitor Thiamet-G (TMG) and glucose to enrich for O-GlcNAcylation. Then the cell lysates were immunoprecipitated with anti-Flag antibodies and immunoblotted with RL2 antibodies. *B*, higher-energy collision dissociation (HCD) mass spectrometry (MS) identified that Ser-4 and Ser-8 are O-GlcNAcylated. *C*, quantitation of (*A*). *D*, the potential O-GlcNAc sites Ser-4 and Ser-8 are conserved. Quantitation in *D* was done with a Student’s *t* test. ∗ indicates *p* < 0.05.

To find out the sites of O-GlcNAcylation, we first tested the O-GlcNAcylation on exogenously overproduced Flag-RPA2 and found it is O-GlcNAcylated (Fig. 2*A*). Then Flag-RPA2-transfected cellular lysates were IPed with anti-Flag antibodies, and the immunoprecipitates were subject to MS analysis (Fig. 2*B*). HCD MS identified a peptide modified by two O-GlcNAc residues and fragmentation suggested that Ser4 and Ser8 were modified (Fig. 2*B*) (Table S1). Then we treated cells with the OGA inhibitor (Thiamet-G, TMG) plus glucose to enrich for O-GlcNAcylation (22, 23) (abbreviated as TMG + Glu), and observed that mutations at Ser4 and Ser8 (S4A/S8A) greatly diminished RL2 signals, suggesting that these two sites were modified (Fig. 2, *A* and *C*). Sequence alignment shows that RPA2 Ser4 and Ser8 are conserved (Fig. 2*D*). Taken together, our results suggest that RPA2 is O-GlcNAcylated and the major modification site could be Ser-4 and Ser-8.

### RPA2 O-GlcNAcylation at S4/S8 antagonizes pS4/S8 upon replication stress

Phosphorylation of RPA2 at Ser4/Ser8 has been known to be crucial for RPA2 function, as PIKK-mediated RPA2 phosphorylation of Ser4 and Ser8 regulates the cell cycle and DNA damage (24) and replication fork restart (25). Therefore, we investigated whether O-GlcNAcylation antagonizes RPA2 Ser4/Ser8 phosphorylation.

**Figure 3:**
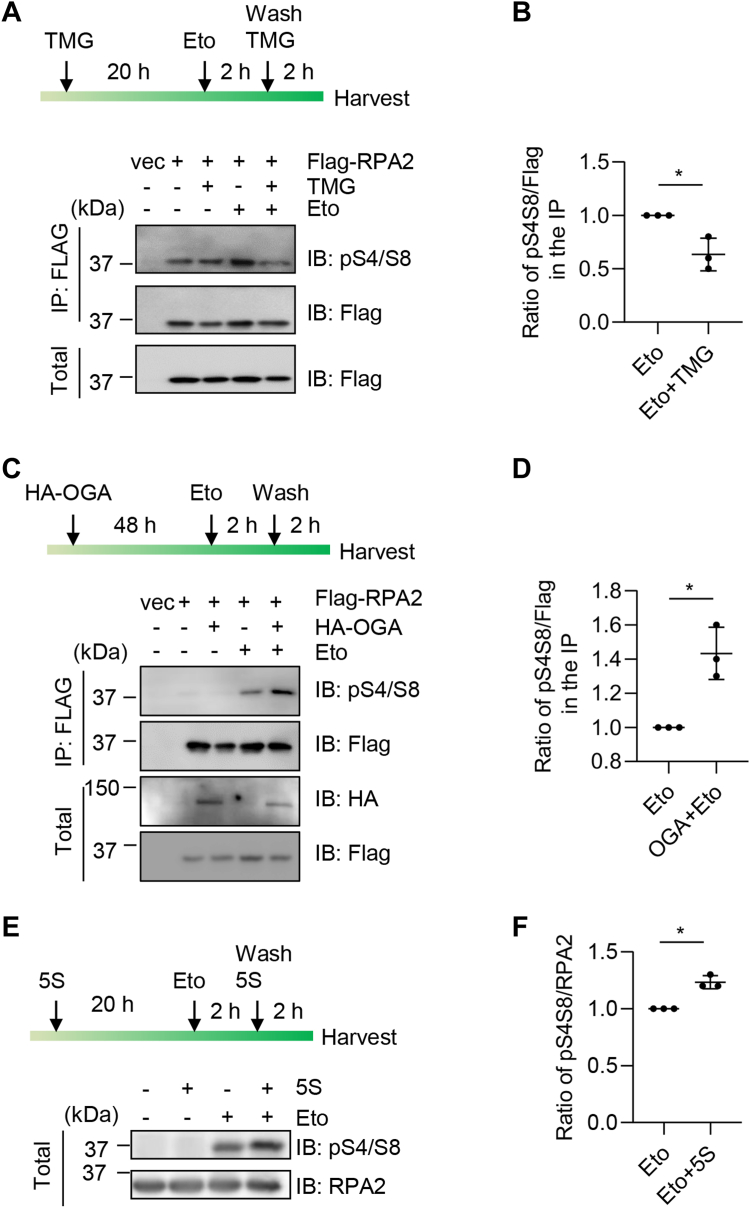
**RPA2 O-GlcNAcylation at S4/S8 antagonizes phosphorylation at S4/S8.***A*, 293T cells were transfected with vectors and Flag-RPA2 plasmids, treated or not treated with Etoposide (Eto) and TMG as shown in the diagram. The lysates were immunoblotted with the antibodies indicated. *B*, quantitation of the results in (*A*). *C*, 293T cells were transfected with vectors, Flag-RPA2 and HA-OGA plasmids. The lysates were immunoblotted with the antibodies indicated. *D*, quantitation of the results in (*C*). *E*, cells were treated with Eto and 5S (acetyl-5S-GlcNAc, OGT inhibitor) to examine its effect on endogenous RPA2. *F*, quantitation of the results in (*E*). A Student’s *t* test was used in (*B*) (*D*) and (*F*) 10. ∗ indicates *p* < 0.05.

To test this, we transfected Flag-RPA2 plasmids into 293T cells. Cells were first treated with TMG to enrich for O-GlcNAcylation, then treated with Eto to induce replication stress. Eto was subsequently washed off, and cells were collected after 2 h to examine for the Ser4/Ser8 phosphorylation levels (Fig. 3*A*). As shown in Figure 3, *A* and *B*, Ser4/Ser8 phosphorylation was significantly decreased in the TMG/Eto lane compared to the Eto only lane. We also overproduced HA-OGA plasmids, and examined its effect on pSer4/Ser8 (Fig. 3*C*), and Ser4/Ser8 phosphorylation was significantly elevated upon HA-OGA overexpression (Fig. 3, *C* and *D*). Moreover, we used acetyl-5S-GlcNAc (5S, OGT inhibitor) to examine its effect on endogenous RPA2 (Fig. 3*E*), and 5S treatment enhanced pSer4/Ser8 levels in the Eto-treated group (Fig. 3, *E* and *F*), suggesting that RPA2 O-GlcNAcylation counteracts Ser4/Ser8 phosphorylation.

### RPA2 O-GlcNAcylation inhibits CHK1 activation during replication stress

**Figure 4:**
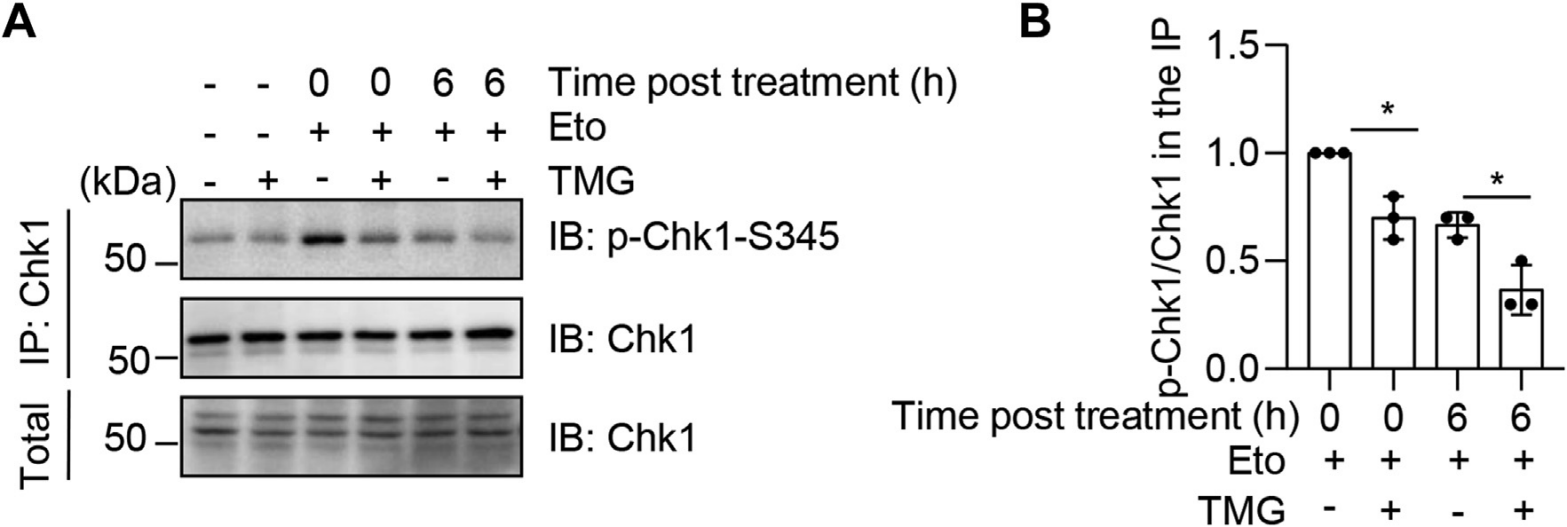
**RPA2 O-GlcNAcylation inhibits CHK1 activation.***A*, cells were treated or untreated with 20 μM Etoposide for 2 h and released for 0 or 6 h, and treated or not treated with TMG. The cell lysates were immunoprecipitated with anti-Chk1 antibodies and immunoblotted with the antibodies indicated. *B*, quantitation of the results in (*A*). A Student’s *t* test was used in (*B*). ∗ indicates *p* < 0.05.

Eto is known to be a chemotherapeutic inhibitor of topoII (26). Replication stress activates ATR, which phosphorylates and activates Chk1, a key step in transmitting DNA damage signals to downstream replication checkpoint effector proteins (27, 28). Previous studies reveal that DNA-PKcs phosphorylation of RPA2 Ser4/Ser8 contributes to Chk1 activation (24) and replication fork restart (25). To determine whether O-GlcNAcylation of RPA2 regulates the activation of Chk1, we incubated the cells with TMG or not, treated cells with Eto for 2 h, and released them for 6 h (Fig. 4*A*). Cell extracts were immunoprecipitated with anti-Chk1 antibodies and the immunoprecipitates were immunoblotted with anti-Chk1-pS345 antibodies, indicative of Chk1 activation (Fig. 4, *A* and *B*). We found that TMG attenuated the levels of Chk1 phosphorylation (Fig. 4, *A* and *B*), suggesting that RPA2 O-GlcNAcylation inhibits Chk1 activation.

### RPA2 O-GlcNAcylation inhibits phospho-H2AX resolution

**Figure 5:**
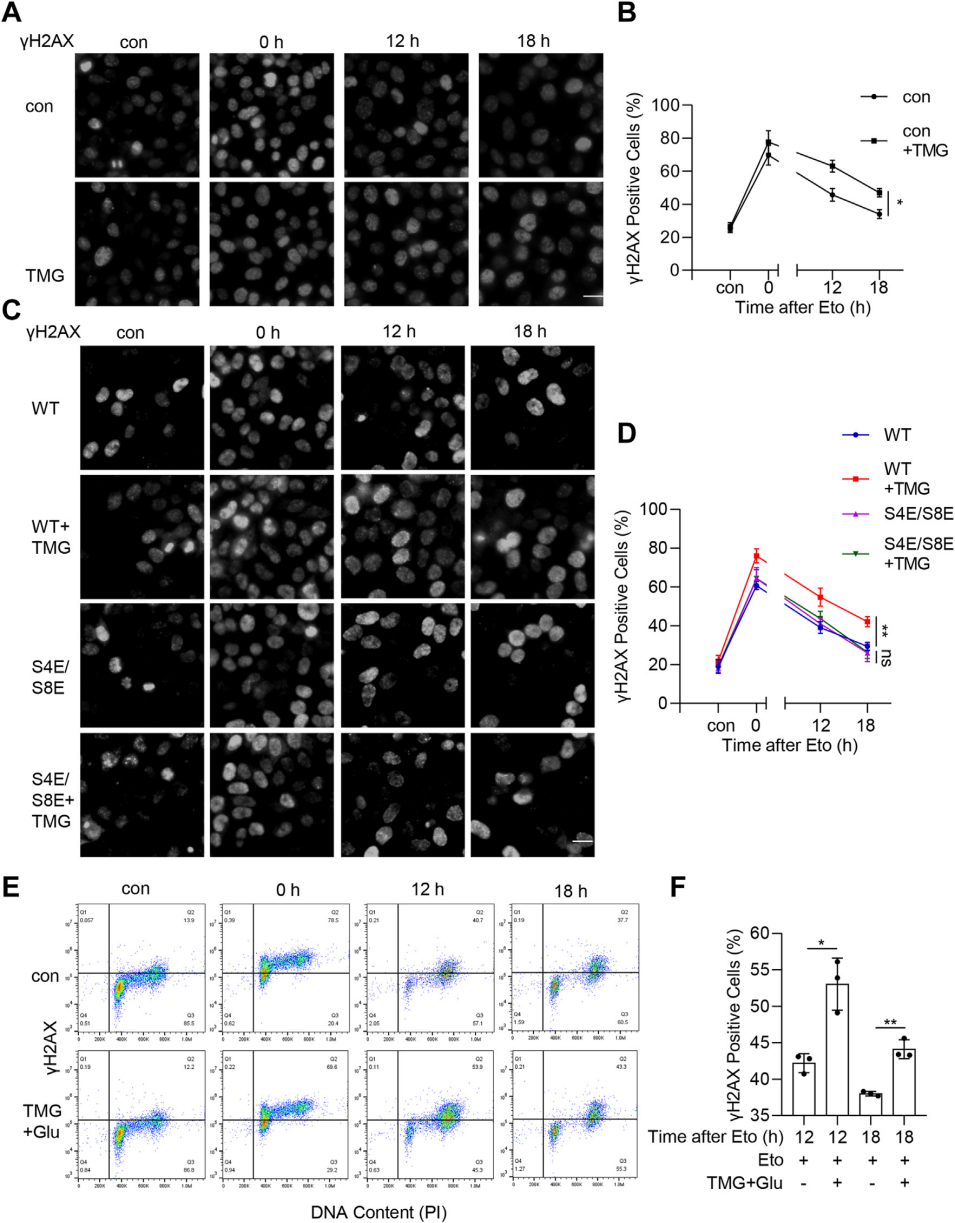
**RPA2 O-GlcNAcylation inhibits γH2AX resolution.***A*, cells were treated or untreated with TMG, and treated (or not treated) with 20 μM Etoposide for 2 h and released for 0 h, 12 h or 18 h. The cells were then stained with anti-γH2AX antibodies and DAPI. *B*, quantitation of the results in (*A*). *C*, cells were transfected with RPA2-WT, or -S4E/S8E plasmids, and then treated as in (*A*). *D*, quantitation of the results in (*C*). These experiments were repeated three times, with n = 50. Scale bar = 10 μm. *E*, cells were treated or untreated with TMG and Glucose, and treated or not treated with 20 μM Etoposide for 2 h and released for indicated times. Then the γH2AX positive cells and DNA content were measured by flow cytometric. *F*, quantitation of the results in (*E*). Quantitation in (*B*) and (*D*) was done with a two-way Anova, in (*F*) with a Student’s *t* test. ∗ indicates *p* < 0.05; ∗∗ indicates *p* < 0.01.

As the phosphorylation of RPA2 plays an important role in response to replication stress, we tested the role of RPA2 O-GlcNAcylation in replication stress. The level of γH2AX is a measure of double-strand breaks (DSBs) and sites of excessive ssDNA at stalled forks and therefore, can be used to monitor replication stress-induced damage and subsequent repair (29, 30). We analyzed γH2AX signals. After TMG treatment, cells showed more persistent γH2AX signals after being released from Eto, as assayed by immunofluorescence microscopy (Fig. 5, *A* and *B*). To tease out whether the effect of TMG was indeed due to RPA2 O-GlcNAcylation, we generated RPA2-S4E/S8E (2E) mutants (Fig. 5, *C* and *D*). And the results showed that TMG did not significantly affect the γH2AX signals in 2E mutations, suggesting that RPA2 glycosylation is vital for the phenotype. Flow cytometry also demonstrated that TMG treatment led to more γH2AX signals after release from Eto (Fig. 5, *E* and *F*). These results indicate that RPA2 O-GlcNAcylation antagonizes phosphorylation and causes the accumulation of damage after Eto release, indicating its role in maintaining replication checkpoint arrest.

### RPA2 O-GlcNAcylation inhibits etoposide-induced S and G_2_/M checkpoint arrest

**Figure 6:**
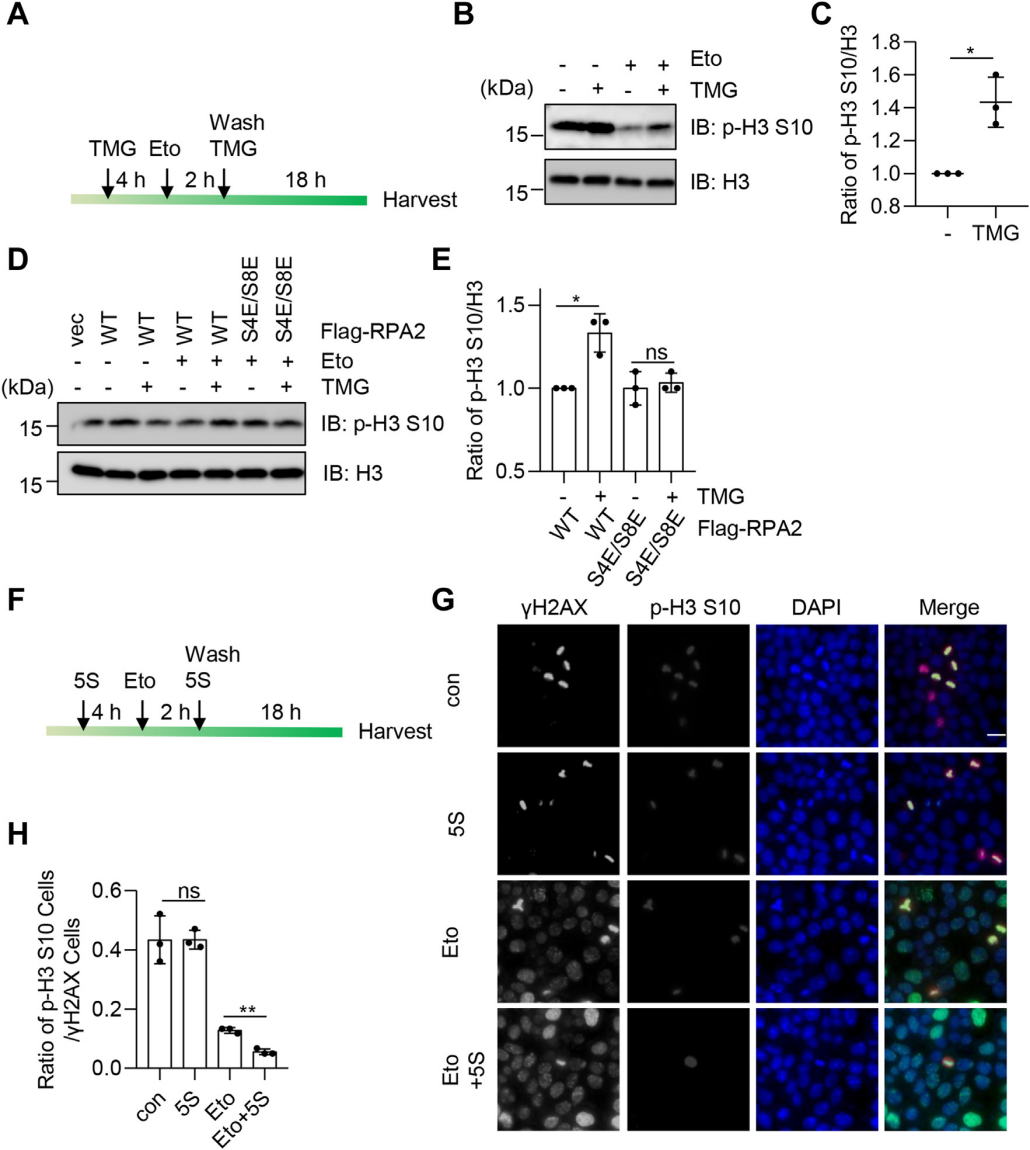
**RPA2 O-GlcNAcylation inhibits Etoposide-induced S and G2/M checkpoint arrest.***A* and *B*, cells were treated or not treated with Etoposide for 2 h and released for 18 h. Histone extraction was carried out to examine p-H3 S10 levels after being treated with TMG. *C*, quantitation of the results in (*B*). *D* and *E*, cells were transfected with RPA2-WT, or -S4E/S8E plasmids, then treated as described in (*A*). *E*, quantitation of the results in (*D*). *F*–*H*, cells were treated or untreated with Etoposide for 2 h and released for 18 h and treated or untreated with 5S. The cells were then stained with anti-γH2AX antibodies, anti-p-H3 S10 antibodies and DAPI. *H*, quantitation of the results in (*G*). These experiments were repeated three times, with n = 60. Scale bar = 10 μm. A Student’s *t* test was used in (*C*), (*E*) and (*H*). ∗ indicates *p* < 0.05; ∗∗ indicates *p* < 0.01. ns, non-specific.

It is known that cells are particularly vulnerable to genome destabilizing and cytotoxic effects of DNA damage when DNA is replicated in the S phase and during chromosome segregation in the M phase (16, 31). The Chk1 activation defect and persistent γH2AX seen with TMG treatment suggest that DNA damage accumulates when these cells fail to arrest in the S phase in response to replication stress. To determine if RPA2 Ser4/Ser8 O-GlcNAcylation is also important to prevent damaged cells from progressing into the M phase, we first treated cells with TMG to enrich for O-GlcNAcylation, then with Eto to induce replication stress, and subsequently released them for 18 h to allow cells to enter mitosis (Fig. 6*A*). We then analyzed the level of phosphorylation of histone H3 Ser10 (pS10-H3), an M phase marker. The Eto/TMG treatment significantly increased histone H3 pSer10 levels (Fig. 6, *B* and *C*), suggesting that RPA2 O-GlcNAcylation did not arrest cells in the S and G_2_/M checkpoint, thus allowing cells to continue into mitosis. We also used the RPA2-2E mutant (Fig. 6, *D* and *E*), and TMG treatment did not significantly affect H3 pSer10 levels, indicating that RPA2 O-GlcNAcylation is vital for the effect.

Moreover, we also used the OGT inhibitor 5S and immunofluorescence microscopy to analyze histone H3-pS10 and γH2AX (Fig. 6*F*). Our results showed that upon Eto treatment, 5S markedly decreased the ratio of H3-pS10-positive over γH2AX-positive cells (Fig. 6, *G* and *H*). These results indicate that RPA2 O-GlcNAcylation inhibits G_2_/M checkpoint activation, thus allowing inappropriate mitotic entry in the presence of unresolved DNA damage.

## Discussion

In this work, we present evidence that RPA2 interacts with OGT and is O-GlcNAcylated at Ser4/Ser8, two well-established phosphorylation sites. We demonstrate that O-GlcNAcylation antagonizes phosphorylation, and inhibits Chk1 activation, γH2AX resolution, and Eto-induced S and G2/M checkpoint arrest.

The role of RPA2 phosphorylation has long been appreciated. The hierarchical phosphorylation of Ser33-Ser23/Ser29-Ser4/Ser8 not only involves the intricate interplay between the different kinases but also renders RPA2 to function at the nexus of the RPA complex and regulate its function (16, 32). Here we demonstrate RPA2 O-GlcNAcylation at Ser4/Ser8, suggesting that O-GlcNAc plays a negative role during replication stress. In light of the recent view that replication stress could be a driver for cellular aging (33), O-GlcNAc may exert its role in aging (34) *via* replication stress.

RPA is recently shown to be able to phase separately (35). Specifically, phosphorylation of the N-terminus of RPA2 is required for RPA assembly into condensates and phase separation (35). In light of the inhibitory role of O-GlcNAc in phase separation (36, 37, 38, 39), it is plausible that RPA2 O-GlcNAc suppresses condensate formation by counteracting phosphorylation.

Besides RPA2, RPA1 is also shown to be modified. RPA1 is SUMOylated at K449/K577 to promote the recruitment of the downstream recombinase Rad51 to the DNA damage sites for HR (40). RPA1 is acetylated by PCAF/GCN5 at Lys-163 to stabilize the RPA1/XPA complex in nucleotide excision repair (41). RPA1 is also crotonylated at K88/K379/K595 and functions in HR (42). In a recent chemoproteomic screen for the O-GlcNAc proteome in HR, RPA1 is also found to be O-GlcNAcylated at Thr-590 (43). We did a preliminary experiment and found that RPA1 is indeed O-GlcNAcylated (data not shown), suggesting that OGT has more substrates in replication and replication stress.

OGT has many substrates in replication, but its role in replication stress is less studied. The data so far suggest that O-GlcNAcylated proteins promote replication progression, such as MCM(9), Top2A(11), histone H4S47 (10) and PARG (4). The work we present here suggests that OGT inhibits checkpoint activation during replication stress, which could contribute to tumorigenesis.

## Experimental procedures

### Cell culture, antibodies, and plasmids

Cells were purchased from ATCC. The cell lines were validated using STR profiling and were free from *mycoplasma* contamination for all experiments. Antibodies: anti-HA (Bethyl Laboratories, A190-108A); anti-FLAG (Sigma, F1084); anti-β-actin (Sigma, A5441); anti-RPA2 (CST, E8X5P); anti-phospho-RPA2 (S4/S8) (Thermo Fisher, A300-245A); anti-OGT (Abcam, AB96718); anti-RL2 (Abcam, AB2739); anti-Chk1 (Santa cruz, 2G11D5); anti-p-Chk1 (S345) (CST, 2341S); anti-phospho-Histone H2A X (Ser139) (Merck, 05-636); anti-phospho-Histone H3 (S10) (Abcam, AB177218). *RPA2-S4/S8A* plasmids were generated using specific primers (sequences available upon request) following the manufacturer's instructions (ClonExpress Ultra One Step Cloning Kit, Vazyme C115).

### Immunoprecipitation (IP) and immunoblotting (IB) assays

IP and IB experiments were performed as described before (22, 44). The following primary antibodies were used for IB: anti-HA (1:1000), and anti-FLAG M2 (Sigma) (1:1000), anti-β-actin (1:3000), anti-OGT (1:1000), anti-RPA2 (1:1000), anti-phospho-RPA2 (S4/S8) (1:1000), anti-RL2 (1:1000), anti-Chk1 (1:1000), anti-p-Chk1 (S345) (1:1000), anti-phospho-Histone H3 (S10) (1:1000). Peroxidase-conjugated secondary antibodies were from JacksonImmuno Research. Blotted proteins were visualized using the ECL detection system (Amersham). Signals were detected by a LAS-4000, and quantitatively analyzed by densitometry using the Multi Gauge software (Fujifilm). All western blots were repeated at least three times.

### Cell culture treatment

Chemical utilization: Thiamet-G (TMG) (OGA inhibitor) was used at 5 μM for 24 h; acetyl-5S-GlcNAc (5S) (OGT inhibitor) was used at 100 μM (prepared at 50 mM in DMSO) for 24 h; Etoposide was used at 20 μM for 2 h and released at different times.

### Click chemistry

Cells were transfected with Flag-RPA2 plasmids, then treated with 200 μM Ac_3_6AzGlcNAc and 5 μM Thiamet-G (Sigma-Aldrich) for 24 h. Collected cells were lysed with 150 mM lysis buffer (150 mM NaCl, 1 M Tris-HCl (pH 7.5), 0.5 M EDTA, 10% NP-40) containing a protease inhibitor cocktail (Roche) for 1 h at 4 °C. Next, cell lysates were cleared using centrifugation (4 °C; 12,000 rpm; 10 min). The supernatant was incubated with 50 μM DBCO-PEG4-Biotin from Duyouyou Biotechnology, 8 mM urea, 10 m M Hepes (pH 7.9), and Halt protease & phosphatase inhibitor cocktail (100×) from Thermo Fisher Scientific, then the pull-down complex was isolated by streptavidin-coupled beads and subjected to Western blotting analysis.

### Indirect immunofluorescence

Indirect immunofluorescence staining was performed as described before (44). Dilutions of primary antibodies were 1:1000 for anti-phospho-Histone H2A X (Ser139), and 1:1000 for anti-phospho-Histone H3 (S10) antibodies. Cell nuclei were stained with DAPI. All immunofluorescence experiments were repeated three times, with 50 or 60 cells per experiment.

### Flow cytometry

HeLa cells were treated or untreated with TMG and glucose, and treated or untreated with 20 μM Etoposide for 2 h and released for indicated times. Cells were harvested with 0.25% trypsin, fixed in 70% ice-ethanol overnight, and permeabilized by 0.25% Triton X-100 on ice for 15 min. Cells were incubated in 50 μg/ml propidium iodide and 100 μg/ml RNase A at room temperature for 30 min, followed by DNA content analysis using BD Accuri C6. The DNA content was analyzed using FlowJo software (https://www.flowjo.com/).

### Higher-energy collision dissociation (HCD) mass spectrometry (MS)

For identification of O-GlcNAcylation by mass spec, proteins isolated by gel electrophoresis were digested with trypsin (Promega) in 100 mM NH_4_HCO_3_ pH 8. The LC-MS/MS analysis was performed on an Easy-nLC 1000 II HPLC (Thermo Fisher Scientific) coupled to a Q-Exactive HF mass spectrometer (Thermo Fisher Scientific). Peptides were loaded on a pre-column (75 μm ID, 6 cm long, packed with ODS-AQ 10 μm, 120 Å beads from YMC Co, Ltd) and further separated on an analytical column (75 μm ID, 12 cm long, packed with Luna C18 1.9 μm 100 Å resin from Welch Materials) using a linear gradient from 100% buffer A (0.1% formic acid in H_2_O) to 30% buffer B (0.1% formic acid in acetonitrile), 70% buffer A in 70 min at a flow rate of 200 nl/min. The top 20 most intense precursor ions from each full scan (resolution 120,000) were isolated for HCD MS2 (resolution 15,000; normalized collision energy 27) with a dynamic exclusion time of 60 s. Precursors with a charge state of 1+, 7+ or above, or unassigned, were excluded.

The software pFind 3 (45, 46) was used to identify O-GlcNAcylated peptides by setting a variable modification of 203.0793 Da at S, T. The mass accuracy of precursor ions and that of fragment ions were both set at 20 ppm. The results were filtered by applying a 1% FDR cutoff at the peptide level and a minimum of one spectrum per peptide. The MS2 spectra were annotated using pLabel (47).

## Data availability

The mass spectrometry proteomics data have been deposited to the ProteomeXchange Consortium (https://proteomecentral.proteomexchange.org) *via* the iProX partner repository (48, 49) with the dataset identifier PXD053321.

## Supporting information

This article contains supporting information (45, 46).

## Conflict of interest

The authors declare that they have no conflicts of interest with the contents of this article.
