# Supplementary material for: O-GlcNAcylation of RPA2 at S4/S8 antagonizes phosphorylation and regulates checkpoint activation during replication stress[image]
Source: J Biol Chem. 2024 Nov 2;300(12):107956. doi: 10.1016/j.jbc.2024.107956 (PMC11647514; doi:10.1016/j.jbc.2024.107956)
Supplement: Supplemental Table S1 [file mmc1.pdf]

Site	Spectral count	Peptide count	Final score	Peptide
**S4, S8**	**1**	**1**	**0.069**	**WNSGFESYGSSSYGGAGGYTQ SPGGFGSPAPSQAEKK**

Chi, H., Liu, C., Yang, H. et al. Comprehensive identification of peptides in tandem mass spectra using an efficient open search engine. Nat Biotechnol 36, 1059–1061 (2018).

Shao G, Cao Y, Chen Z, et al. How to use open-pFind in deep proteomics data analysis?—A protocol for rigorous identification and quantitation of peptides and proteins from mass spectrometry data[J]. Biophysics reports, 2021, 7(3): 207.
